# 2,2′-Dichloro-1,1′-[(butane-1,4-diyldi­oxy)bis­(nitrilo­methyl­idyne)]dibenzene

**DOI:** 10.1107/S1600536808024355

**Published:** 2008-08-06

**Authors:** Zong-Li Ren, Wen-Kui Dong, Wen-Juan Bai, Xue-Ni He, Li Wang

**Affiliations:** a School of Chemical and Biological Engineering, Lanzhou Jiaotong University, Lanzhou 730070, People’s Republic of China

## Abstract

The mol­ecule of the title compound, C_18_H_18_Cl_2_N_2_O_2_, lies across a crystallographic inversion centre and adopts an *E* configuration with respect to the azomethine C=N bond. The imino group is coplanar with the aromatic ring. Within the mol­ecule, the planar units are parallel, but extend in opposite directions from the dimethyl­ene bridge. In the crystal structure, the title compound exhibits a layer packing structure *via* weak π–π stacking inter­actions [inter­molecular plane-to-plane distances between adjacent aromatic rings are 3.461 (3) Å]. Mol­ecules in each layer are linked by inter­molecular C—H⋯O hydrogen-bonding inter­actions.

## Related literature

For related literature, see: Collison & Fenton (1996 ▶); Dong, He *et al.* (2007 ▶); Dong, Duan *et al.* (2007 ▶); Dong *et al.* (2008 ▶); Liu *et al.* (2008 ▶); Lu *et al.* (2006 ▶); Mandal *et al.* (1996 ▶); Shi *et al.* (2007 ▶); Yu *et al.* (2007 ▶, 2008 ▶).
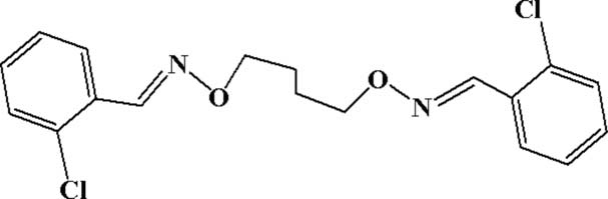


## Experimental

### 

#### Crystal data

C_18_H_18_Cl_2_N_2_O_2_
*M* *_r_* = 365.24
Monoclinic, 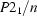

*a* = 4.5296 (5) Å
*b* = 6.6231 (8) Å
*c* = 29.963 (2) Å
β = 92.526 (2)°
*V* = 898.02 (16) Å^3^
*Z* = 2
Mo *K*α radiation
μ = 0.37 mm^−1^
*T* = 298 (2) K
0.48 × 0.28 × 0.13 mm

#### Data collection

Bruker SMART 1000 diffractometer
Absorption correction: multi-scan (*SADABS*; Sheldrick, 1996 ▶) *T*_min_ = 0.841, *T*_max_ = 0.953
4304 measured reflections
1531 independent reflections
1310 reflections with *I* > 2σ(*I*)
*R*_int_ = 0.057

#### Refinement

*R*[*F* ^2^ > 2σ(*F* ^2^)] = 0.074
*wR*(*F* ^2^) = 0.164
*S* = 1.10
1531 reflections
109 parameters
H-atom parameters constrained
Δρ_max_ = 0.21 e Å^−3^
Δρ_min_ = −0.29 e Å^−3^

### 

Data collection: *SMART* (Siemens, 1996 ▶); cell refinement: *SAINT* (Siemens, 1996 ▶); data reduction: *SAINT*; program(s) used to solve structure: *SHELXS97* (Sheldrick, 2008 ▶); program(s) used to refine structure: *SHELXL97* (Sheldrick, 2008 ▶); molecular graphics: *SHELXTL* (Sheldrick, 2008 ▶); software used to prepare material for publication: *SHELXTL*.

## Supplementary Material

Crystal structure: contains datablocks global, I. DOI: 10.1107/S1600536808024355/om2252sup1.cif

Structure factors: contains datablocks I. DOI: 10.1107/S1600536808024355/om2252Isup2.hkl

Additional supplementary materials:  crystallographic information; 3D view; checkCIF report

## Figures and Tables

**Table 1:** Hydrogen-bond geometry (Å, °)

*D*—H⋯*A*	*D*—H	H⋯*A*	*D*⋯*A*	*D*—H⋯*A*
C8—H8⋯O1^i^	0.93	2.66	3.581 (5)	171

Symmetry code: (i) 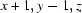.

**Fig. 1.**
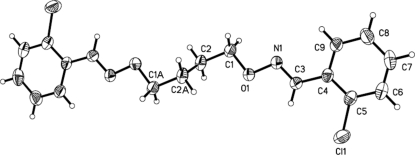
The molecular structure of the title compound with atom numbering scheme [Symmetry codes: -x, -y + 2, -z + 1]. Displacement ellipsoids for non-hydrogen atoms are drawn at the 30% probability level.

**Fig. 2.**
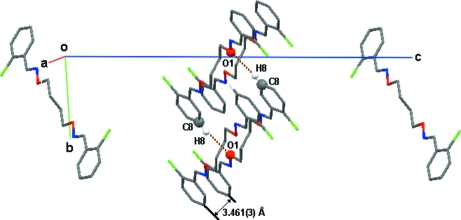
The packing diagram of the title compound showing intermolecular hydrogen bonds and π-π stacking interactions.

## supplementary crystallographic information

### Comment

Schiff bases are an important class of compounds which can be used in a variety of studies such as organic synthesis, catalyst, drug design, material science and life science and so on (Collison, *et al.*, 1996; Mandal, *et al.*, 1996). In the past decades, a continuing attention has been drawn to the Schiff bases derived from benzaldehyde or salicylaldehyde and their metal complexes for the investigation of luminescent properties which could be finely tuned by different substituent groups bonded to the phenolic ring (Lu *et al.*, 2006; Yu *et al.*, 2007; Yu *et al.*, 2008). Here, in continuation of our previous studies (Dong, Duan *et al.*, 2007; Shi, *et al.*, 2007), we report the synthesis and X-ray structure of a new Schiff base bisoxime compound 2,2'-dichloro-1,1'-[butane-1,4-diyldioxybis(nitrilomethylidyne)]dibenzene.

The crystal structure of the title compound is built up by only the C_18_H_18_Cl_2_N_2_O_2_ molecules, in which all bond lengths are in normal ranges. The molecule, as shown in Fig. 1, lies across a crystallographic inversion centre (symmetry code: -*x*, -*y*, -*z*) and adopts an E configuration with respect to the azomethine C=N bond. The imino group is coplanar with the aromatic ring. Within the molecule, the planar units are parallel, with the distance 1.480 (4) Å [intra-molecular plane-to-plane distance], but extend in opposite directions from the dimethylene bridge. In the crystal structure, (Fig. 2) the title compound exhibits a layer packing structure *via* weak π-π stacking interactions [inter-molecular plane-to-plane distances between adjacent aromatic rings is 3.461 (3) Å]. Molecules in each layer are linked by intermolecular C8—H8···O1 hydrogen bonding interactions [C8···O1, 3.581 (5) Å].

### Experimental

2,2'-Dichloro-1,1'-[butane-1,4-diyldioxybis(nitrilomethylidyne)]dibenzene was synthesized according to an analogous method reported earlier (Dong, He *et al.*, 2007; Dong, *et al.*, 2008; Liu, *et al.*, 2008). To an ethanol solution (3 ml) of 2-chloro-benzaldehyde (281.1 mg, 2.00 mmol) was added an ethanol solution (2 ml) of 1, 4-bis(aminooxy)butane (120.2 mg, 1.00 mmol). The mixture solution was stirred at 328 K for 4 h. When cooled to room temperature, the precipitate was filtered, and washed successively with ethanol and hexane, respectively. The product was dried under vacuum and purified with recrystallization from ethanol to yield 219.8 mg of the title compound. Yield, 60.1%. mp. 334–335 K. Anal. Calc. for C_18_H_18_Cl_2_N_2_O_2_: C, 59.19; H, 4.97; N, 7.67. Found: C, 59.22; H, 5.03; N, 7.58.

Colorless needle-shaped single crystals suitable for X-ray diffraction studies were obtained after several weeks by slow evaporation from an ethyl-acetate/acetone mixed solution of the title compound.

### Refinement

Non-H atoms were refined anisotropically. H atoms were treated as riding atoms with distances C—H = 0.97 (CH_2_), 0.93 Å (CH), and *U*_iso_(H) = 1.2 *U*_eq_(C) and 1.5 *U*_eq_(O).

### Crystal data

**Table:**

C_18_H_18_Cl_2_N_2_O_2_	*F*_000_ = 380
*M**_r_* = 365.24	*D*_x_ = 1.351 Mg m^−^^3^
Monoclinic, *P*2_1_/*n*	Mo *K*α radiation λ = 0.71073 Å
Hall symbol: -P 2yn	Cell parameters from 2364 reflections
*a* = 4.5296 (5) Å	θ = 3.2–28.2º
*b* = 6.6231 (8) Å	µ = 0.37 mm^−^^1^
*c* = 29.963 (2) Å	*T* = 298 (2) K
β = 92.526 (2)º	Needle, colorless
*V* = 898.02 (16) Å^3^	0.48 × 0.28 × 0.13 mm
*Z* = 2	

### Data collection

**Table:**

Bruker SMART 1000 diffractometer	1531 independent reflections
Radiation source: fine-focus sealed tube	1310 reflections with *I* > 2σ(*I*)
Monochromator: graphite	*R*_int_ = 0.057
*T* = 298(2) K	θ_max_ = 25.0º
φ and ω scans	θ_min_ = 1.4º
Absorption correction: multi-scan(SADABS; Sheldrick, 1996)	*h* = −5→5
*T*_min_ = 0.841, *T*_max_ = 0.953	*k* = −7→5
4304 measured reflections	*l* = −35→34

### Refinement

**Table:**

Refinement on *F*^2^	Secondary atom site location: difference Fourier map
Least-squares matrix: full	Hydrogen site location: inferred from neighbouring sites
*R*[*F*^2^ > 2σ(*F*^2^)] = 0.074	H-atom parameters constrained
*wR*(*F*^2^) = 0.164	*w* = 1/[σ^2^(*F*_o_^2^) + (0.0452*P*)^2^ + 1.0643*P*] where *P* = (*F*_o_^2^ + 2*F*_c_^2^)/3
*S* = 1.10	(Δ/σ)_max_ = 0.001
1531 reflections	Δρ_max_ = 0.21 e Å^−^^3^
109 parameters	Δρ_min_ = −0.29 e Å^−^^3^
Primary atom site location: structure-invariant direct methods	Extinction correction: none

### Special details

**Table:**

Geometry. All e.s.d.'s (except the e.s.d. in the dihedral angle between two l.s. planes) are estimated using the full covariance matrix. The cell e.s.d.'s are taken into account individually in the estimation of e.s.d.'s in distances, angles and torsion angles; correlations between e.s.d.'s in cell parameters are only used when they are defined by crystal symmetry. An approximate (isotropic) treatment of cell e.s.d.'s is used for estimating e.s.d.'s involving l.s. planes.
Refinement. Refinement of *F*^2^ against ALL reflections. The weighted *R*-factor *wR* and goodness of fit *S* are based on *F*^2^, conventional *R*-factors *R* are based on *F*, with *F* set to zero for negative *F*^2^. The threshold expression of *F*^2^ > σ(*F*^2^) is used only for calculating *R*-factors(gt) *etc*. and is not relevant to the choice of reflections for refinement. *R*-factors based on *F*^2^ are statistically about twice as large as those based on *F*, and *R*- factors based on ALL data will be even larger.

### Fractional atomic coordinates and isotropic or equivalent isotropic displacement parameters (Å^2^)

**Table:**

	*x*	*y*	*z*	*U*_iso_*/*U*_eq_	
Cl1	0.8257 (3)	0.7688 (2)	0.71001 (4)	0.0939 (5)	
N1	0.4408 (7)	0.6370 (5)	0.57897 (10)	0.0570 (8)	
O1	0.2543 (6)	0.7961 (4)	0.56512 (8)	0.0607 (7)	
C1	0.0979 (8)	0.7356 (6)	0.52466 (11)	0.0598 (9)	
H1A	−0.0273	0.6204	0.5304	0.072*	
H1B	0.2370	0.6968	0.5025	0.072*	
C2	−0.0885 (8)	0.9111 (6)	0.50759 (13)	0.0643 (10)	
H2A	−0.2160	0.8650	0.4828	0.077*	
H2B	−0.2141	0.9551	0.5311	0.077*	
C3	0.5650 (8)	0.6708 (6)	0.61688 (12)	0.0570 (9)	
H3	0.5233	0.7892	0.6321	0.068*	
C4	0.7733 (7)	0.5252 (5)	0.63692 (11)	0.0521 (8)	
C5	0.9101 (8)	0.5566 (6)	0.67866 (12)	0.0591 (9)	
C6	1.1129 (9)	0.4202 (7)	0.69728 (13)	0.0697 (11)	
H6	1.2038	0.4454	0.7252	0.084*	
C7	1.1776 (10)	0.2505 (8)	0.67465 (16)	0.0820 (13)	
H7	1.3133	0.1587	0.6871	0.098*	
C8	1.0431 (11)	0.2122 (7)	0.63302 (16)	0.0826 (13)	
H8	1.0867	0.0946	0.6177	0.099*	
C9	0.8444 (9)	0.3496 (6)	0.61452 (12)	0.0636 (10)	
H9	0.7562	0.3241	0.5865	0.076*	

### Atomic displacement parameters (Å^2^)

**Table:**

	*U*^11^	*U*^22^	*U*^33^	*U*^12^	*U*^13^	*U*^23^
Cl1	0.1045 (9)	0.1019 (10)	0.0731 (7)	0.0144 (8)	−0.0213 (6)	−0.0275 (7)
N1	0.0621 (18)	0.0562 (17)	0.0522 (17)	0.0094 (15)	−0.0026 (14)	0.0042 (14)
O1	0.0694 (16)	0.0559 (15)	0.0555 (14)	0.0125 (12)	−0.0105 (12)	−0.0013 (12)
C1	0.063 (2)	0.064 (2)	0.0514 (19)	−0.0040 (19)	−0.0059 (15)	0.0049 (18)
C2	0.056 (2)	0.075 (3)	0.061 (2)	−0.0034 (19)	−0.0106 (17)	0.018 (2)
C3	0.064 (2)	0.057 (2)	0.0498 (19)	0.0071 (18)	−0.0003 (16)	−0.0012 (16)
C4	0.0524 (19)	0.060 (2)	0.0436 (17)	0.0034 (17)	0.0046 (14)	0.0085 (16)
C5	0.059 (2)	0.073 (2)	0.0451 (18)	−0.0021 (19)	0.0045 (15)	0.0073 (18)
C6	0.061 (2)	0.092 (3)	0.055 (2)	0.003 (2)	−0.0034 (17)	0.020 (2)
C7	0.074 (3)	0.089 (3)	0.083 (3)	0.022 (3)	0.000 (2)	0.033 (3)
C8	0.097 (3)	0.071 (3)	0.080 (3)	0.030 (3)	0.011 (2)	0.013 (2)
C9	0.074 (2)	0.069 (2)	0.0483 (19)	0.010 (2)	0.0036 (17)	0.0047 (18)

### Geometric parameters (Å, °)

**Table:**

Cl1—C5	1.742 (4)	C3—H3	0.9300
N1—C3	1.265 (4)	C4—C5	1.387 (5)
N1—O1	1.402 (4)	C4—C9	1.387 (5)
O1—C1	1.434 (4)	C5—C6	1.388 (5)
C1—C2	1.513 (5)	C6—C7	1.351 (6)
C1—H1A	0.9700	C6—H6	0.9300
C1—H1B	0.9700	C7—C8	1.387 (7)
C2—C2^i^	1.506 (8)	C7—H7	0.9300
C2—H2A	0.9700	C8—C9	1.379 (5)
C2—H2B	0.9700	C8—H8	0.9300
C3—C4	1.460 (5)	C9—H9	0.9300
			
C3—N1—O1	111.8 (3)	C5—C4—C3	121.8 (3)
N1—O1—C1	108.0 (3)	C9—C4—C3	121.0 (3)
O1—C1—C2	108.6 (3)	C4—C5—C6	121.7 (4)
O1—C1—H1A	110.0	C4—C5—Cl1	120.5 (3)
C2—C1—H1A	110.0	C6—C5—Cl1	117.8 (3)
O1—C1—H1B	110.0	C7—C6—C5	119.6 (4)
C2—C1—H1B	110.0	C7—C6—H6	120.2
H1A—C1—H1B	108.4	C5—C6—H6	120.2
C2^i^—C2—C1	114.0 (4)	C6—C7—C8	120.4 (4)
C2^i^—C2—H2A	108.8	C6—C7—H7	119.8
C1—C2—H2A	108.8	C8—C7—H7	119.8
C2^i^—C2—H2B	108.8	C9—C8—C7	119.6 (4)
C1—C2—H2B	108.8	C9—C8—H8	120.2
H2A—C2—H2B	107.7	C7—C8—H8	120.2
N1—C3—C4	120.4 (3)	C8—C9—C4	121.3 (4)
N1—C3—H3	119.8	C8—C9—H9	119.3
C4—C3—H3	119.8	C4—C9—H9	119.3
C5—C4—C9	117.3 (3)		
			
C3—N1—O1—C1	−173.4 (3)	C3—C4—C5—Cl1	2.6 (5)
N1—O1—C1—C2	−176.2 (3)	C4—C5—C6—C7	−0.9 (6)
O1—C1—C2—C2^i^	66.7 (5)	Cl1—C5—C6—C7	178.0 (3)
O1—N1—C3—C4	−178.7 (3)	C5—C6—C7—C8	0.1 (7)
N1—C3—C4—C5	−179.1 (4)	C6—C7—C8—C9	0.7 (7)
N1—C3—C4—C9	1.5 (6)	C7—C8—C9—C4	−0.7 (7)
C9—C4—C5—C6	0.8 (5)	C5—C4—C9—C8	−0.1 (6)
C3—C4—C5—C6	−178.6 (3)	C3—C4—C9—C8	179.4 (4)
C9—C4—C5—Cl1	−178.0 (3)		

Symmetry codes: (i) −*x*, −*y*+2, −*z*+1.

### Hydrogen-bond geometry (Å, °)

**Table:**

*D*—H···*A*	*D*—H	H···*A*	*D*···*A*	*D*—H···*A*
C8—H8···O1^ii^	0.93	2.66	3.581 (5)	171

Symmetry codes: (ii) *x*+1, *y*−1, *z*.
